# Emerging zoonotic diseases in Southeast Asia in the period 2011–2022: a systematic literature review

**DOI:** 10.1080/01652176.2023.2300965

**Published:** 2024-01-16

**Authors:** Thanh Trung Nguyen, Thi Ngan Mai, Sinh Dang-Xuan, Hung Nguyen-Viet, Fred Unger, Hu Suk Lee

**Affiliations:** a Faculty of Veterinary Medicine, Vietnam National University of Agriculture, Hanoi, Vietnam; b International Livestock Research Institute, Regional Office for East and Southeast Asia, Hanoi, Vietnam; c College of Veterinary Medicine, Chungnam National University, Daejeon, South Korea

**Keywords:** Emerging zoonotic diseases, pig, poultry, ruminant, companion animal, wildlife, southeast Asia, prevalence

## Abstract

As COVID-19 has shown, pandemics and outbreaks of emerging infections such as Zika, Nipah, monkeypox and antimicrobial-resistant pathogens, especially emerging zoonotic diseases, continue to occur and may even be increasing in Southeast Asia. In addition, these infections often result from environmental changes and human behaviour. Overall, public health surveillance to identify gaps in the literature and early warning signs are essential in this region. A systematic review investigated the prevalence of emerging zoonotic diseases over 11 years from 2011 to 2022 in Southeast Asia to understand the status of emerging zoonotic diseases, as well as to provide necessary actions for disease control and prevention in the region. During the 2011–2022 period, studies on pigs, poultry, ruminants, companion animals and wildlife in Southeast Asia were reviewed thoroughly to assess the quality of reporting items for inclusion in the systematic review. The review was performed on 26 studies of pigs, 6 studies of poultry, 21 studies of ruminants, 28 studies of companion animals and 25 studies of wildlife in Southeast Asia, which provide a snapshot of the prevalence of the emerging zoonotic disease across the country. The findings from the review showed that emerging zoonotic diseases were prevalent across the region and identified a few zoonotic diseases associated with poultry, mainly stemming from Cambodia and Vietnam, as high priority in Southeast Asia.

**Clinical relevance**: Appropriate prevention and control measures should be taken to mitigate the emerging zoonotic diseases in Southeast Asia.

## 1. Introduction

Livestock production is critical to human nutrition and health in low- and middle-income countries (LMICs) (Milton 2003). These animals play important roles in society, providing income and food, but also clothing, building materials, fertilizer, and draught power. However, the presence of endemic and emerging diseases, as well as other factors, impact livestock negatively, jeopardizing their contributions.

Many new science-based policy reports continue to focus on the global public health emergency caused by the COVID-19 pandemic following the fast spread of the infectious virus of possible zoonotic origin (Wu et al. 2022). As of 19 August 2023, about 7 million people had died of COVID-19 worldwide. This pandemic has had a staggering effect on the global economy and countless other effects in both developing and developed countries. The cost of controlling and containing it has reached several trillion US dollars.

Around 60% of all human diseases are zoonotic and 75% of all emerging diseases are considered zoonoses (Woolhouse and Gowtage-Sequeria 2005). Historically, the emergence of new human diseases from animals has been associated with major societal change. For example, during the neolithic transition from hunter-gathering to agricultural societies, humans lived shorter lives, ate less and poorer-quality foods, were smaller in size and were sicker than their hunter-gatherer ancestors (Larsen 1995). With the advent of agriculture, the dramatic rise in population and the settlement of people near their waste led to increases in human disease; the domestication of animals led to livestock pathogens jumping species into people, where they became the probable cause of diseases (Morand et al. 2014). These new emerging disease outbreaks followed rapid intensification of agricultural practices and systems to meet increased demand for animal protein, and dramatic changes in the ways animals were kept or farmed, often without proper precautionary measures (Jones et al. 2013). This was a demand-driven process, associated with increasing wealth and allowing people to consume more animal-source foods.

Although the origin of COVID-19 is currently not known, it may be associated with wildlife harvest, trade practices and the intensification of wildlife farming (Whitfort 2021). The latter is actively occurring in several countries, in which wildlife breeding and farming ventures have been established during the recent past. Although wealthy consumers in these countries tend to prefer wild-caught animals, the meat from these farms is often consumed by the rapidly growing middle-class in several parts of the world.

The socioeconomic crises caused by the recent outbreaks of COVID-19 (2019) (Huang et al. 2020); (Gaffar Sarwar and Mesfer Al 2021), African swine fever (2018) (Liu et al. 2020), avian influenza (2004) in Asia (Webster et al. 2005) with the most recent human case of avian influenza A(H3N8) and A(H5N1) was reported in 2023 from China and Cambodia, respectively (Venkatesan 2023). These diseases have served to heighten awareness of the wide-ranging negative impacts of infectious diseases on human health, food safety, livestock trade, and livelihoods of poor farming communities. All these diseases may have a wildlife reservoir and may also involve domestic animals. In addition to these outbreaks, continued losses of livestock belonging mainly to smallholders in Southeast Asia due to other transboundary animal and emerging diseases have clearly revealed major weaknesses in the public health and veterinary services. This literature review aims to understand the current status of emerging zoonotic diseases in Southeast Asia, as well as to provide necessary actions for disease control and prevention in the region.

## 2. Materials and methods

### 2.1. Protocol and eligibility criteria

As summarized in Table 1, developing the protocol for the search and evaluation of the articles was included in the objective, data source, and inclusion and exclusion criteria. Only articles in English and online databases were considered for this review. In the first screening, the titles and abstracts were examined thoroughly to see if they were suitable for the present review. The second screening examined the quality of the full publication based on different inclusion and exclusion criteria (Table 1). All procedures were performed independently by all the authors (TTN, TNM, XSD and HSL). Each article was classified as ‘Yes’ or ‘No’ for inclusion. The first reason for the exclusion of articles that passed the first screening was the lack of information on how the selection of farms and individuals was carried out. Additionally, the second reason was poor random selection at the farm level and of individual animals such as targeted sampling of individuals showing symptoms of the disease. If there was a conflict between the four reviewers, the final decision was made after a discussion among them.

**Table 1.** Establishing inclusion and exclusion criteria in this study.

Inclusion criteria	Exclusion criteria
First screening	
Original English research articles (Peer-reviewed)	Study not conducted in Southeast Asia
Published 2011–2022 (by 11th March)	Articles about avian influenza^a^
Presenting prevalence, outbreaks data of zoonotic/livestock diseases	Review articles
Second screening	
Cross-sectional study	
Random selection of individuals	
Clear description of methods and results	

^a^ *Avian influenze are defined as a regional priority transboundary animal diseases.*

### 2.2. Searching strategy and syntaxes

In the context of the present review, the term ‘emerging zoonotic diseases’ is to refer to diseases that are either newly recognized, newly introduced or newly evolved, or have recently and rapidly changed in incidence or expansion in their geographical, host or vector range and transmitted under natural conditions from vertebrate animals to humans (Stevenson and Halpin 2021). Databases are organized collections of resources of articles. The authors searched for relevant articles in the PubMed, Web of Science and Science Direct databases. The key syntaxes were divided into three topics – (i) (livestock OR swine OR pig OR cattle OR buffalo OR sheep OR goat OR poultry OR duck OR chicken OR pets OR dogs OR cats OR rats); AND (ii) (Brunei OR Cambodia OR Indonesia OR Laos PDR OR Malaysia OR Myanmar OR Philippines OR Singapore OR Thailand OR Timor-Leste OR Vietnam OR Southeast Asia); AND (iii) (zoonoses OR zoonotic diseases).

The full lists of titles and abstracts were imported into Endnote (version X7), and duplicates were manually identified and removed. The last search was performed on 11 March 2022. To ensure that the search strategy captured all relevant articles, we checked that known key articles were included in the results. In the second screening, we also cross-checked the grey literature on the reference lists of the articles against our search results to make sure we did not miss any relevant articles. The time span under review was 2011–2022. The management programs used were Endnote and Excel 365.

### 2.3. Data collection

The data extraction template included the authors, publication year, pathogen name, animal species, diagnostic method, study area, sample size, number of positive samples, prevalence, and 95% confidence interval (CI). In cases where several methods were applied to one sample, the highest prevalence was released. If the 95% CI of the prevalence or the number of positive animals was absent in an article, this information was derived using the data presented in the article. The data from eligible publications were reviewed and extracted into a Microsoft Excel file. Lastly, the extracted dataset was independently cross-checked against each original article by the same four authors (TTN, TNM, XSD and HSL).

### 2.4. Synthesis of results

Descriptive statistics were summarized by species like pigs, poultry, ruminants, companion animal and wildlife with the following information: pathogen, country, year of sampling, sample size, % positive, diagnostic test, 95% CI, author, and year.

## 3. Results

### 3.1. Zoonotic diseases in Southeast Asia

#### 3.1.1. Article finding and screening

**Figure 1.**
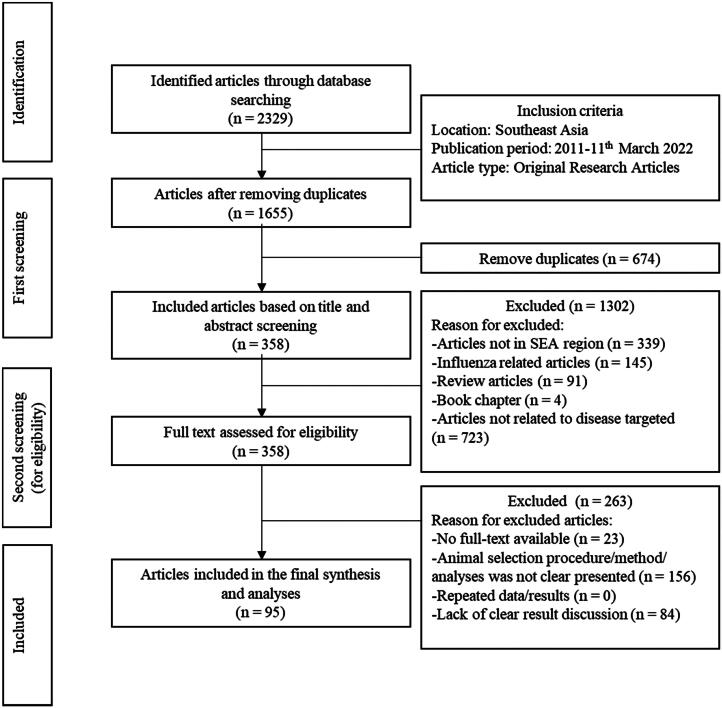
Schematic flow chart of the literature selection for the review on zoonotic diseases in Southeast Asia.

A total of 2,329 articles were retrieved from PubMed (*n* = 1043), Web of Science (*n* = 994) and Science Direct (*n* = 292). In the first screening, 674 duplicates were identified and removed, and 1,302 publications were excluded due to not conducting in southeast Asia (*n* = 339) or due to focusing on influenza (*n* = 145). Also eliminated were review articles (*n* = 91), book chapters (*n* = 4) and articles not related to the targeted diseases (*n* = 723). Thus, a total of 358 articles were included in the full-text assessment (Figure 1). The list of articles (including titles, authors, abstracts and years of publication) is attached in the Annex (Excel) file.

Subsequently, a total of 358 full-text articles were assessed in the second screening, where 263 articles were excluded because of the full text being unavailable (*n* = 23), the animal selection procedure being unclear (*n* = 156), or the results not being presented clearly (*n* = 84). Thus, 95 publications were included in the final qualitative synthesis (Figure 1).

#### 3.1.2. Descriptions of 358 articles

**Figure 2.**
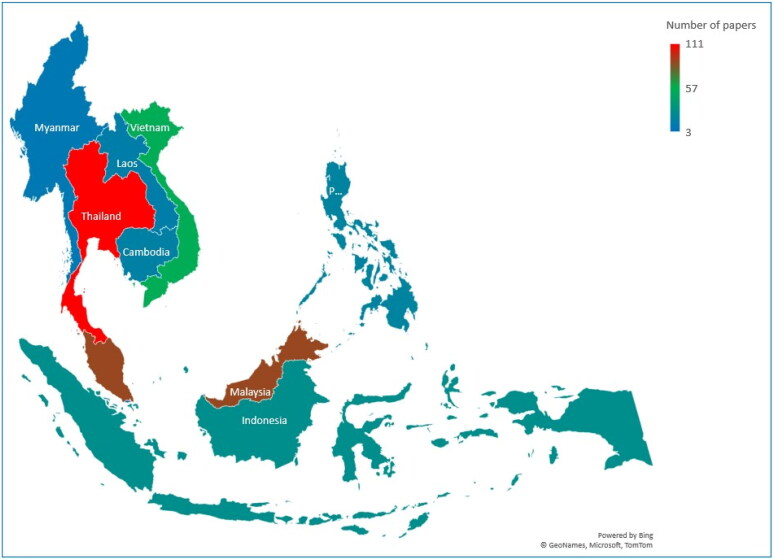
Geographical distribution of studies on zoonotic pathogens in Southeast Asia.

**Figure 3.**
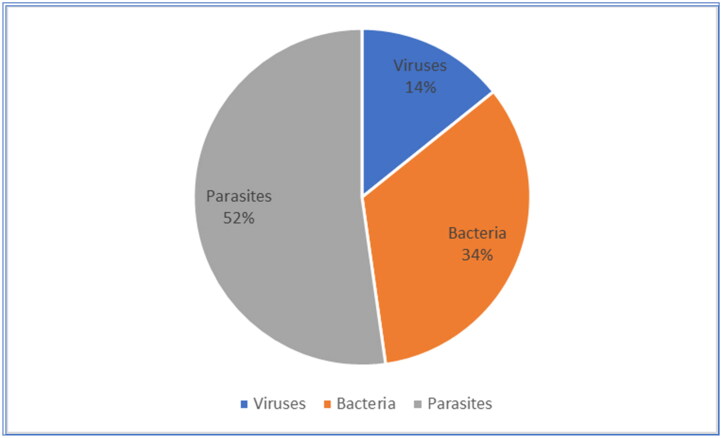
Focus of studies on zoonotic pathogens in Southeast Asia.

Most of the papers found were on studies conducted in Thailand (111/358), followed by Malaysia (89/358), Vietnam (54/358), Indonesia (28/358), Cambodia (17/358), the Philippines (19/358), Laos (15/358), Myanmar (10/358) and Singapore (3/358), as depicted in Figure 2. Of these articles, 12/358 were multi-country studies (Figure 2), and 14% (51/358) focused on viral pathogens, 34% (120/358) on bacterial pathogens and 52% (187/358) on parasitic pathogens (Figure 3).

### 3.2. Zoonotic diseases associated with domestic pigs in Southeast Asia

The qualitative synthesis yielded 26 published ­articles reporting on 35 studies related to pigs (Table 2). The three areas of research covered under this topic were bacteria, viruses and parasites. Specifically, diseases associated with pigs were as follows: Japanese encephalitis, Hepatitis E, rotavirus A, kobuviruses, *Campylobacter*, *Streptococcus suis*, trichinellosis, erysipelas, Salmonella, cryptosporidiosis, cysticercosis/taeniasis, leptospirosis and toxoplasmosis. Out of these, nine studies (30% of the total) were conducted in Cambodia, including 7 studies on parasites, 1 study on bacteria (*Campylobacter*) and 1 study on viruses (Japanese encephalitis). The largest number of studies was conducted in Vietnam (14 studies, or 39%). Among them, there were 3 studies on parasites, 7 studies on bacteria and 4 studies on viruses. Subsequently, there were 4 studies on bacteria, 1 study on viruses and 1 study on parasites in Cambodia. There were 2 studies (5%) on viruses in Laos, and only 1 study each (3%) in Indonesia (parasite), Malaysia (viruses) and the Philippines (parasites). In summary, these data highlighted the importance of zoonotic diseases originating from pigs, with Cambodia and Vietnam having the highest numbers of studies in Southeast Asia.

**Table 2.** List of studies focusing on zoonotic diseases in domestic pigs in Southeast Asia.

Pathogen	Country	Year of sampling	Sample size	% positive	Diagnostic test	95% CI	References
*Ascaris suum*	Cambodia	2011	30	13.3	FECT	4.4–31.6	(Inpankaew et al. 2015)
*Gnathostoma hispidum*	Cambodia	2011	30	6.67	FECT	1.2–23.5	(Inpankaew et al. 2015)
Capillaria	Cambodia	2011	30	6.67	FECT	1.2–23.5	(Inpankaew et al. 2015)
*Fasciolopsis buski*	Cambodia	2011	30	30	FECT	15.4–49.6	(Inpankaew et al. 2015)
Taenia	Cambodia	2019	242	11.2	ELISA	7.5–15.8	(Söderberg et al. 2021)
Trichinella	Cambodia	2019	242	2.5	ELISA	0.9–5.4	(Söderberg et al. 2021)
Japanese encephalitis	Cambodia	2019	197	92.9	ELISA	88.1–95.9	(Henriksson et al. 2021)
Campylobacter	Cambodia	2011–2013	162	6.79	Culture	3.6–12.1	(Osbjer et al. 2016)
Campylobacter	Cambodia	2011–2013	162	11.73	PCR	7.4–17.9	(Osbjer et al. 2016)
*Taenia solium*	Cambodia	2014–2015	620	4.68	ELISA	3.2–6.7	(Adenuga et al. 2018)
*Cryptosporidium parvum*	Indonesia	2019	205	6.34	PCR	3.6–10.8	(Resnhaleksmana et al. 2021)
Japanese encephalitis	Laos	2008–2009	727	74.70	HI	71.5–77.9	(Conlan et al. 2012)
Hepatitis E	Laos	2008–2009	722	21.10	ELISA	18.1–24.0	(Conlan et al. 2012)
Japanese encephalitis	Malaysia	2015–2016	45	46.70	ELISA	0.724–1.652	(Kumar et al. 2018b)
Trichinella	Philippines	2017	555	0.54	ELISA	0.11–1.57	(Lagrimas et al. 2021)
Salmonella	Thailand	2014	82	41.46	Culture	30.9–52.9	(Patchanee et al. 2016)
Leptospira	Thailand	2013–2016	152	3.95	Culture	1.6–8.8	(Kurilung et al. 2017)
Leptospira	Thailand	2013–2016	152	7.89	PCR	4.3–13.7	(Kurilung et al. 2017)
Hepatitis E	Thailand	2014–2015	3478	1.58	PCR	1.2–2.1	(Intharasongkroh et al. 2017)
Cryptosporidium	Thailand	2015–2016	245	20.82	PCR	16.1–26.6	(Thathaisong et al. 2020)
*Streptococcus suis*	Thailand	2016–2017	88	85.23	PCR	75.7–91.6	(Boonyong et al. 2019)
Japanese encephalitis	Vietnam	2009	43	100	ELISA	89.8–100	(Lindahl et al. 2013)
Campylobacter	Vietnam	2012	61	57.38	Culture	44.1–69.7	(Carrique-Mas et al. 2014)
Hepatitis E	Vietnam	2012	774	19.12	PCR	16.4%–22.1%	(Berto et al. 2018)
Rotavirus A	Vietnam	2012	730	32.74	PCR	29.4–36.3	(Pham et al. 2014)
Leptospira	Vietnam	2016	1959	8.17	MAT	6.99–9.47	(Lee et al. 2017)
Leptospirosis	Vietnam	2017	2000	21.05	MAT	19.28–22.90	(Lee et al. 2019)
Japanese encephalitis	Vietnam	2017	2000	73.45	ELISA	73.73–86.41	(Lee et al. 2019)
Taenia	Vietnam	2018	399	10.28	PCR	7.6–13.8	(Nguyen, Dermauw, et al. 2020)
Kobuviruses	Vietnam	2012–2014	682	29.33	PCR	26.0–32.9	(Van Dung et al. 2016)
Hepatitis E	Vietnam	2013–2014	293	8.19	PCR	5.3–11.9	(Berto et al. 2018)
Burkholderia pseudomallei	Vietnam	2018–2019	1125	6.3	ELISA	5–7.9	(Norris et al. 2020)
Streptococcus suis	Vietnam	2019–2020	174	2.30	PCR	0.6–5.8	(Nguyen, Luu, et al. 2021)
Hepatitis E	Vietnam	2018–2019	475	58.53	ELISA	53.95–62.70	(Lee et al. 2020)
Trichinella	Vietnam	N/A	558	5.6	ELISA	3.9–7.9	(Thi et al. 2013)

CI: confidence interval; N/A: not available; PCR: Polymerase Chain Reaction; ELISA: Enzyme-linked immunosorbent assay; MAT: Modified agglutination test; FECT: formalin-ether concentration technique; HI: hemagglutination-inhibition.

### 3.3. Zoonotic diseases associated with poultry in Southeast Asia

As depicted in Table 3, six of the articles reporting on 10 studies that were included in the qualitative synthesis were related to poultry. Out of these, 4 studies (40%) were conducted in Cambodia, including 4 studies on bacteria *(Campylobacter*). Most of the studies were conducted in Vietnam (5 studies, or 50%), comprising 4 studies on bacteria and 1 study on parasites. Subsequently, there was 1 study (10%) on viruses in Thailand. Specifically, the study investigating *Campylobacter* found the highest prevalence in poultry in Cambodia. Taken together, Salmonella, *Streptococcus suis*, Echinostome and *Opisthorchis viverrine* were the most common pathogen. The data indicated that only a few zoonotic diseases associated with poultry, mainly stemming from Cambodia and Vietnam, were identified as high priority in Southeast Asia.

**Table 3.** List of studies focusing on zoonotic diseases in poultry in Southeast Asia.

Pathogen	Country	Year of sampling	Species	Sample size	% positive	Diagnostic test	95% CI	References
Campylobacter	Cambodia	2011–2013	Chicken	353	24.65	Culture	20.3–29.5	(Osbjer et al. 2016)
Campylobacter	Cambodia	2011–2013	Chicken	353	56.09	PCR	50.7–61.3	(Osbjer et al. 2016)
Campylobacter	Cambodia	2011–2013	Duck	101	4.95	Culture	1.8–11.7	(Osbjer et al. 2016)
Campylobacter	Cambodia	2011–2013	Duck	101	23.76	PCR	16.1–33.4	(Osbjer et al. 2016)
Echinostome	Thailand	2011–2012	Duck	90	56.67	PCR	45.8–66.9	(Saijuntha et al. 2013)
Campylobacter	Vietnam	2012	Chicken	100	24.00	Culture	16.3–33.8	(Carrique-Mas et al. 2014)
Campylobacter	Vietnam	2012	Duck	83	18.07	Culture	10.8–28.4	(Carrique-Mas et al. 2014)
*Opisthorchis viverrine*	Vietnam	2013–2015	Duck	178	34.30	PCR	20.7–40.4	(Dao et al. 2016)
Salmonella	Vietnam	2011	Chicken	1000	45.9	Culture	42.8–49.0	(Ta et al. 2012)
*Streptococcus suis*	Vietnam	2018	Chicken	59	33.90	Culture	19–80	(Nhung et al. 2020)

CI: confidence interval; PCR: Polymerase Chain Reaction.

### 3.4. Zoonotic diseases associated with ruminants in Southeast Asia

As shown in Table 4, twenty-one of the articles that were included in the qualitative synthesis were related to ruminants as described in 35 studies. Out of these, 11 studies (31%) were conducted in Thailand and another 11 (31%) in Laos; 4 (11%) in Malaysia; 3 (9%) in Indonesia; and 2 (6%) each in Cambodia, the Philippines and Vietnam. Diseases associated with ruminants were as follows: *Campylobacter*, Q fever, brucellosis, enterohaemorrhagic *E. coli*, listeriosis, bovine tuberculosis, chlamydiosis, cryptosporidiosis, cysticercosis/taeniasis, salmonellosis, toxoplasmosis, fascioliasis, and giardiasis.

**Table 4.** List of studies focusing on zoonotic diseases in ruminants in Southeast Asia.

Pathogen	Country	Year of sampling	Species	Sample size	% positive	Diagnostic test	95% CI	References
Campylobacter	Cambodia	2011–2013	Cattle	207	0.97	Culture	0.17–3.8	(Osbjer et al. 2016)
Campylobacter	Cambodia	2011–2013	Cattle	207	5.31	PCR	2.8–9.6	(Osbjer et al. 2016)
Brucellosis	Indonesia	2019–2020	Dairy	588	5.78	Rose Bengal test	4.1–8.1	(Yanti et al. 2021)
Brucellosis	Indonesia	2019–2020	Dairy	588	5.10	Complement fixation test	3.5–7.3	(Yanti et al. 2021)
Escherichia coli O157:H7	Indonesia	2013	Cattle	238	6.30	Culture	3.7–10.4	(Suardana et al. 2017)
Coxiellosis	Laos	2016–2017	Goat	1458	4.10	ELISA	3–5	(Burns et al. 2018)
Brucella	Laos	2016–2017	Goat	1458	1.40	ELISA	0.8–2.2	(Burns et al. 2018)
Trichostrongylid	Laos	2010	Goat	14	92.86	Culture	64.2–99.6	(Sato et al. 2014)
Trichostrongylid	Laos	2010	Cattle	11	27.27	Culture	7.3–60.6	(Sato et al. 2014)
Q fever	Laos	2013–2015	Cattle	526	2.47	ELISA	1.4–4.3	(Douangngeun et al. 2016)
Brucellosis	Laos	2013–2015	Cattle	526	0.57	ELISA	0.15–1.8	(Douangngeun et al. 2016)
T. saginata	Laos	2006	Bovine	905	46.40	ELISA	43.2–49.7	(Vongxay et al. 2012)
Q-fever	Laos	2006	Bovine	905	4.00	ELISA	2.7–5.3	(Vongxay et al. 2012)
Leptospirosis	Laos	2006	Bovine	905	3.10	ELISA	1.9–4.2	(Vongxay et al. 2012)
Tuberculosis	Laos	2006	Bovine	905	1	ELISA	0.3–1.6	(Vongxay et al. 2012)
Brucellosis	Laos	2006	Bovine	905	0.20	ELISA	0.0–0.5	(Vongxay et al. 2012)
Cryptosporidium	Malaysia	2008–2009	Calves	120	23.33	PCR	21.5–32.7	(Muhid et al. 2011)
Fascioliasis	Malaysia	2017–2018	Cattle	308	14.61	Microscopic	11.0–19.2	(Ahmad-Najib et al. 2021)
Bartonella bovis	Malaysia	2013	Cattle	304	3.29	PCR	1.7–6.2	(Kho et al. 2015)
Leptospirosis	Malaysia	2013	Cattle	420	81.7	MAT	63.5– 80.1	(Daud et al. 2018)
Coxiella burnetiid	Philippines	2016–2019	Cattle	512	1.37	PCR	0.6–2.9	(Galay et al. 2020)
Coxiella burnetiid	Philippines	2016–2019	Water buffalo	108	2.78	PCR	0.7–8.5	(Galay et al. 2020)
Leptospira	Thailand	2013–2016	Cattle	131	0.76	Culture	0.04–4.8	(Kurilung et al. 2017)
Leptospira	Thailand	2013–2016	Cattle	131	12.21	PCR	7.4–19.4	(Kurilung et al. 2017)
Babesia	Thailand	2016	Cattle	279	30.47	PCR	25.2–36.3	(Jirapattharasate et al. 2017)
Bartonella	Thailand	2021	Buffalo	156	16.03	IFAT	10.65–22.74	(Boonmar et al. 2021)
Coxiella burnetiid	Thailand	2012–2013	Dairy	988	4.55	ELISA	3.4–6.1	(Doung-Ngern et al. 2017)
Coxiella burnetiid	Thailand	2012–2013	Goat	516	3.49	ELISA	2.1–5.6	(Doung-Ngern et al. 2017)
Coxiella burnetiid	Thailand	2012–2013	Sheep	48	2.08	ELISA	0.1–12.5	(Doung-Ngern et al. 2017)
Cryptosporidium	Thailand	2017	Dairy cow	500	7.00	IFAT	5.0–9.7	(Inpankaew et al. 2017)
Cryptosporidium	Thailand	2017	Dairy cow	500	7.60	PCR	5.5–10.4	(Inpankaew et al. 2017)
Fasciola gigantica	Thailand	2010–2012	Cattle	55	67.27	PCR	53.2–79.0	(Phalee and Wongsawad, 2014)
Fasciola	Thailand	2016–2019	Bull	1501	2.47	Microscopic	1.8–3.4	(Kaewnoi et al. 2020)
Fascioliasis	Vietnam	2014–2015	Cattle	572	23.43	Fecal sedimentation	20.1–27.2	(Nguyen et al. 2017)
Giardia duodenalis	Vietnam	2014–2015	Calves	412	13.83	Microscopic	10.8–17.5	(Nguyen et al. 2016)

CI: confidence interval; PCR: Polymerase Chain Reaction; ELISA: Enzyme-linked immunosorbent assay; MAT: Modified agglutination test; FECT: formalin-ether concentration technique; IFAT: indirect fluorescent antibody test.

### 3.5. Zoonotic diseases associated with companion animals in Southeast Asia

As depicted in Table 5, twenty-eight of the articles that were included in the qualitative synthesis were related to companion animals as described in 54 studies. Out of these, 22 studies (47%) were conducted in Thailand. Subsequently, 5 studies (11%) were conducted in Malaysia; 10 studies (22%) in Cambodia; 3 studies (7%) each in the Philippines and Laos; 2 studies (4%) in Indonesia; and 1 (2%) study in Vietnam. Diseases associated with dogs and cats in this review include rabies, pasteurellosis, Q fever, leptospirosis, salmonellosis, roundworms, hookworms, and giardiasis.

**Table 5.** List of studies focusing on zoonotic diseases in companion animals in Southeast Asia.

Pathogen	Country	Year of sampling	Species	Sample size	% positive	Diagnostic test	95% CI	References
Trematodes	Cambodia	2012	Dog	94	2.13	PCR	0.37–8.2	(Schär et al. 2014)
*Giardia duodenalis*	Cambodia	2012	Dog	94	2.13	PCR	0.37–8.2	(Schär et al. 2014)
*Strongyloides stercoralis*	Cambodia	2013–2016	Dog	29	75.86	PCR	56.1–89.0	(Jaleta et al. 2017)
*A. ceylanicum*	Cambodia	2012	Dog	90	94.44	PCR	87.0–98.0	(Inpankaew et al. 2014)
*Giardia duodenalis*	Cambodia	2011	Dog	50	4.00	FECT	0.7–14.6	(Inpankaew et al. 2015)
Entamaeba	Cambodia	2011	Dog	50	2.00	FECT	0.1–12.0	(Inpankaew et al. 2015)
*Toxocara canis*	Cambodia	2011	Dog	50	8.00	FECT	2.6–20.1	(Inpankaew et al. 2015)
*Strongyloides stercoralis*	Cambodia	2011	Dog	50	8.00	FECT	2.6–20.1	(Inpankaew et al. 2015)
Echinostomes	Cambodia	2011	Dog	50	18.00	FECT	9.0–31.9	(Inpankaew et al. 2015)
*Toxoplasma gondii*	Cambodia	N/A	Dog	103	50.49	IFAT	40.5–60.4	(Nguyen, Kengradomkij, et al. 2021)
*Toxascaris leonina*	Indonesia	2018–2019	Cat	120	10.83	Microscopic	6.1–18.1	(Rabbani et al. 2020)
*Toxascaris leonina*	Indonesia	2018–2019	Cat	120	18.33	Microscopic	12.1–26.7	(Rabbani et al. 2020)
Rickettsia	Laos	N/A	Dog	60	5.00	PCR	1.3–14.8	(Nguyen et al. 2020)
Brucella	Laos	N/A	Dog	60	15.00	PCR	7.5–27.1	(Nguyen et al. 2020)
Leptospira	Laos	N/A	Dog	60	1.67	PCR	0.09–10.1	(Nguyen et al. 2020)
Ancylostoma	Malaysia	N/A	Dog	82	47.56	Microscopic	41.4–54.95	(Mahdy et al. 2012)
*Rickettsia felis*	Malaysia	2010	Dog	209	2.87	PCR	1.2–6.4	(Mokhtar and Tay, 2011)
*Japanese encephalitis*	Malaysia	2015–2016	Cat	90	5.60	ELISA	8.22–22.98	(Kumar et al. 2018a)
*Japanese encephalitis*	Malaysia	2015–2016	Dog	45	80	ELISA	68.31–91.69	(Kumar et al. 2018a)
*Toxoplasma gondii*	Malaysia	2013–2014	Dog	222	23.42	ELISA	17.8–29.2	(Watanabe et al. 2020)
Taenia	Philippine	2017	Dog	200	3.00	direct smear, flotation, sedimentation	2.31–3.69	(Urgel et al. 2019)
Ancylostoma	Philippine	2017	Dog	200	38.00	direct smear, flotation, sedimentation	37.3–38.7	(Urgel et al. 2019)
Anaplasma	Philippines	N/A	Dog	248	22.58	PCR	17.6–28.4	(Galay et al. 2018)
Babesia	Philippines	N/A	Dog	248	7.66	PCR	4.8–11.9	(Galay et al. 2018)
Leptospira	Thailand	2013–2016	Dog	58	6.90	Culture	2.2–17.5	(Kurilung et al. 2017b)
Leptospira	Thailand	2013–2016	Dog	58	10.34	PCR	4.3–21.8	(Kurilung et al. 2017b)
*Ancylostoma ceylanicum*	Thailand	2019	Dog	299	26.42	Microscopic	21.6–31.9	(Kladkempetch et al. 2020)
*Ancylostoma ceylanicum*	Thailand	2019	Dog	58	96.55	PCR	87.0–99.4	(Kladkempetch et al. 2020)
Bartonella	Thailand	2021	Cat	513	2.53	PCR	1.36–4.29	(Saengsawang et al. 2021)
Brucella	Thailand	2019	Dog	16	12.5	ELISA	2.2–39.6	(Ngamkala et al. 2020)
*Dirofilaria repens*	Thailand	2019	Dog	8003	0.44	Microscopic	0.3–0.61	(Jitsamai et al. 2021)
*Opisthorchis viverrini*	Thailand	2014	Dog	197	40.1	latex agglutination test	39.4–40.8	(Pumidonming et al. 2017)
*Opisthorchis viverrini*	Thailand	2014	Cat	180	33.89	latex agglutination test	33.2–34.6	(Pumidonming et al. 2017)
Leptospira	Thailand	2014–2018	Dog	370	32.43	Lepto-latex test	27.7–37.5	(Ngasaman et al. 2020)
Leptospira	Thailand	2014–2018	Dog	370	0.54	PCR	0.1–2.2	(Ngasaman et al. 2020)
Leptospira	Thailand	2014–2018	Cat	64	10.94	Lepto-latex test	4.9–21.8	(Ngasaman et al. 2020)
Leptospira	Thailand	2014–2018	Cat	64	7.81	PCR	2.9–18.0	(Ngasaman et al. 2020)
Leptospira	Thailand	2016–2017	Cat	260	6.15	PCR	3.7–10.0	(Sprißler et al. 2019)
Leptospira	Thailand	N/A	Dog	273	4.40	PCR	2.0–6.8	(Altheimer et al. 2020)
Leptospira	Thailand	N/A	Dog	273	0.37	Culture	0.01–1.1	(Altheimer et al. 2020)
Leptospira	Thailand	N/A	Dog	273	12.09	MAT	8.2–16.0	(Altheimer et al. 2020)
Leptospira	Thailand	N/A	Dog	252	44.05	ELISA	37.9–50.2	(Altheimer et al. 2020)
*Toxoplasma gondii*	Thailand	2019	Dog	318	7.86	MAT	4.9–10.8	(Huertas-López et al. 2021)
*Toxoplasma gondii*	Thailand	2019	Cat	321	18.70	MAT	14.4–23.0	(Huertas-López et al. 2021)
Toxoplasmosis	Thailand	2016–2017	Cat	260	6.54	IFAT	4.0–10.4	(Inpankaew et al. 2021)
*Giardia duodenalis*	Vietnam	2016–2017	Dog	209	8.61	Microscopic	5.3–13.5	(Nguyen et al. 2018)
Trichinella	Vietnam	N/A	Dog	125	4.00	ELISA	1.5–9.6	(Thi et al. 2013)

CI: confidence interval; N/A: not available; PCR: Polymerase Chain Reaction; ELISA: Enzyme-linked immunosorbent assay; MAT: Modified agglutination test; FECT: formalin-ether concentration technique; IFAT: indirect fluorescent antibody test.

### 3.6. Zoonotic diseases associated with wildlife in Southeast Asia

This report further investigates the role of wildlife in zoonotic diseases in Southeast Asia. Specifically, 25 of the articles that were included in the qualitative synthesis were related to wildlife as described in 36 studies (Table 6). Out of these, 12 studies (33%) were conducted in Vietnam; 8 studies (22%) in Malaysia; 7 studies (19%) in Thailand; 4 studies (11%) in Laos; 2 studies (6%) in Indonesia; and 1 study (3%) each in Cambodia, Myanmar and the Philippines.

**Table 6.** List of studies focusing on zoonotic diseases in wildlife in Southeast Asia.

Pathogen	Country	Year of sampling	Species	Sample size	% positive	Diagnostic test	95% CI	References
Coronaviruses	Cambodia	2010–2013	Bat	1059	5.76	PCR	4.5–7.4	(Lacroix et al. 2017)
Ebola	Indonesia	2005–2006	Primate	353	18.41	ELISA	14.6–22.9	(Nidom et al. 2012)
Japanese encephalitis	Indonesia	2015–2018	Bat	373	5.63	PCR	3.6–8.6	(Diptyanusa et al. 2021)
Coronaviruses	Laos	2010–2013	Bat	906	3.53	PCR	2.5–5.0	(Lacroix et al. 2017)
*Bandicota indica*	Laos	2020	Rodent	310	31.29	Microscopic	26.2–36.8	(Sithay et al. 2020)
*Bandicota savilei*	Laos	2020	Rodent	310	10.32	Microscopic	7.3–14.4	(Sithay et al. 2020)
*Leopoldamys edwardsi*	Laos	2020	Rodent	310	58.39	Microscopic	52.7–63.9	(Sithay et al. 2020)
Leptospira	Malaysia	2011–2012	Rat	300	6.67	Culture	4.2–10.3	(Benacer et al. 2013)
Trichostrongylus	Malaysia	2016–2017	Macaques	21	52.38	Direct smear, flotation, sedimentation	30.3–73.6	(Choong et al. 2019)
Mycobacterium tuberculosis	Malaysia	2019–2020	Wild boars	30	16.67	ELISA	7.3–33.5	(Lekko et al. 2021a)
Mycobacterium tuberculosis	Malaysia	2019–2020	Wild boars	12	75.00	PCR	46.8–91.1	(Lekko et al. 2021b)
Mycobacterium tuberculosis	Malaysia	2019–2020	Macaques	30	33.33	PCR	19.2–51.2	(Lekko et al. 2021b)
*Taenia taeniaeformis*	Malaysia	2018–2019	Rat	89	28.09	PCR	19.3–38.8	(Tijjani et al. 2020)
Plasmodium	Malaysia	2016	Macaques	103	62.14	PCR	52.0–71.4	(Amir et al. 2020)
Rickettsiae	Malaysia	2008–2011	Rat	95	13.68	PCR	7.8–22.6	(Tay et al. 2014)
Coronaviruses	Myanmar	2016–2018	Bat	759	6.32	PCR	4.7–8.4	(Valitutto et al. 2020)
Campylobacter	Philippines	2015	Bat	91	5.49	PCR	2.0–12.9	(Hatta et al. 2016)
Bartonella	Thailand	2011	Deer	247	3.64	Culture	1.8–7.0	(Pangjai et al. 2018)
Chlamydia	Thailand	2017	Crocodile	138	91.30	PCR	85.0–95.2	(Paungpin et al. 2021)
*Gongylonema neoplasticum*	Thailand	2014	Rat	98	36.80	Microscopic	29.1 − 49.2	(Ribas, Saijuntha, Agatsuma, Thongjun, et al. 2016)
*Raillietina* sp.	Thailand	2014	Rat	98	34.70	Microscopic	25.4 − 45.0	(Ribas, Saijuntha, Agatsuma, Thongjun, et al. 2016)
*Capillaria hepatica*	Thailand	2014	Rat	98	64.29	Microscopic	53.9–73.5	(Ribas, Saijuntha, Agatsuma, Thongjun, et al. 2016)
Salmonella	Thailand	2014	Rat	110	49.09	PCR	39.5–58.7	(Ribas, Saijuntha, Agatsuma, Prantlová, et al. 2016)
Trypanosoma	Thailand	2015	Rat	100	21.00	PCR	13.8–30.5	(Molee et al. 2019)
Kobuviruses	Vietnam	2012–2014	Wild boars	45	42.22	PCR	28.0–57.8	(Van Dung et al. 2016)
*L. interrogans*	Vietnam	2017–2018	Rat	144	11.81	Culture	7.2–18.5	(Koizumi et al. 2019)
*L. interrogans*	Vietnam	2017–2018	Rat	88	26.14	PCR	17.6–36.8	(Koizumi et al. 2019)
Leptospira	Vietnam	2012–2013	Rat	241	18.26	MAT	13.7–23.8	(Loan et al. 2015)
Leptospira	Vietnam	2012–2013	Rat	275	5.82	PCR	3.5–9.5	(Loan et al. 2015)
Hantavirus	Vietnam	2012–2013	Rat	275	6.91	PCR	4.3–10.8	(Van Cuong et al. 2015)
Hantavirus	Vietnam	2006–2009	Rat	1066	5.63	ELISA	4.4–7.2	(Luan et al. 2012)
Bartonella	Vietnam	2010, 2013, 2014, and 2018	Rodent	133	31.58	PCR	23.9–40.3	(Anh et al. 2021)
Rickettsia	Vietnam	2010, 2013, 2014, and 2018	Rodent	133	24.81	PCR	17.9–33.2	(Anh et al. 2021)
Leptospira	Vietnam	2010, 2013, 2014, and 2018	Rodent	133	18.05	PCR	12.1–25.9	(Anh et al. 2021)
Trichinella	Vietnam	2010–2013	Wild boars	62	3.23	PCR	0.8– 4.8	(Thi et al. 2014)
Trichinella	Vietnam	2010–2013	Rat	820	2.80	PCR	13.7–32.3	(Thi et al. 2014)

CI: confidence interval; N/A: not available; PCR: Polymerase Chain Reaction; ELISA: Enzyme-linked immunosorbent assay; MAT: Modified agglutination test.

## 4. Discussion

To the best of our knowledge, this systemic literature review was the first conducted on emerging zoonotic diseases between 2011 and 2022 in Southeast Asia. It is well known that animal brucellosis is endemic in Southeast Asia (Suresh et al. 2022). This disease is associated with economic losses and significant impacts on human health. Brucellosis in humans can present as an acute or chronic infection. Livestock keepers in Timor-Leste rely on small-scale farming systems (Smith et al. 2019). While there have not been any studies published on human brucellosis in this region, it is known that the activities of abattoir workers are associated with a high risk of infection. There is a high rate of abortion during late gestation in cattle and buffalo, and in rural areas, there is also a high rate of abortion in humans and low fertility among farmers. In line with cultural traditions, abortions are blamed on black magic or superstitious powers. Therefore, literature relating to brucellosis in Timor-Leste is limited. The number of cattle is expected to continue to increase worldwide, thus the role and impact of cattle on the future of public health will likely remain compelling in Southeast Asia.

Bovine tuberculosis (TB) is an illness characterized by pneumonia, enlarged lymph nodes and signs of weakness. There are different strains, but *Mycobacterium bovis* is the most common, with most infections occurring in cattle. The organism can also affect other domestic animal species but cattle are the main reservoir for the pathogen and also the main source of infection for humans. In Laos, TB is endemic and is recognized as a major health risk (Lassausaie et al. 2014). However, the current prevalence in humans is unknown. Notably, the literature on tuberculosis in Laos is also very limited. Laotians consume raw meat regularly (Suwansrinon et al. 2007). For example, the national dish, ‘larb’, is prepared by mixing different types of raw meat, blood and intestines. It may be made from raw chicken, beef, duck, fish or pork, is very popular in rural areas, and, as with other dishes, is often made from home-slaughtered animals. In addition, Laotians also often prefer raw meat in large community events. In some areas of Laos, people also consume blood and raw milk. In addition to risks associated with consumption, occupational risks are present for those in close contact with animals in Laos. An example is abattoir workers who are at high risk due to frequent exposure to blood from slaughtered animals in an environment with poor hygiene measures (Suwansrinon et al. 2007).

Among the zoonoses diseases in this review, viral diseases such as Japanese encephalitis can infect pets and be transmitted to humans. Cutaneous contamination with *Leptospira* spp. is also emerging/re-emerging pathogens that can be transmitted by our pets, as well as flu-like illness pathogens such as brucellosis (Chomel 2014). Parasitic and fungal pathogens, such as rickettsia, echinococcosis, giardiasis or sporotrichosis, are also re-emerging or emerging pet-related zoonoses (Rahman et al. 2020). Therefore, this report highlighted the role of small companion animals in zoonotic disease risk in Southeast Asia.

Notably, there is no available information that the government of Brunei conducts surveillance of zoonotic disease. That could be a possible reason that there was no published data available on emerging zoonotic diseases in Brunei. On the contrary, there is publicly evidence that the government of Singapore has a One health framework for the surveillance and reporting of zoonotic disease. However, to date no reported case was recorded through the surveillance system (Lysaght et al. 2016). Furthermore, livestock farming is the principal source of livelihood for most countries in Southeast Asia except Singapore and Brunei (Hassan 2014). As a result, it may be explained that we could find any evidence regarding the zoonotic diseases in Singapore in the period 2011 − 2022.

It should be noted that rabies is incurable and has the highest fatality rate of any zoonosis (Lavan et al. 2017). Rabies was first reported in Indonesia in 1889 and it is currently endemic in 33 provinces. Indonesia’s national strategic plan highlights the importance of control and eradication of the disease as a national priority. Indonesia is identified as having the fourth-highest number of human rabies cases after India, Bangladesh and Myanmar, with 150 − 300 cases reported yearly in Indonesia (World Health Organization 2016). In 2008, Bali experienced an outbreak of rabies leading to many human fatalities (Putra et al. 2013). In response to this outbreak, a mass culling of stray dogs was implemented, along with mass vaccination of owned dogs. Initial efforts did not control the outbreak and by 2010, rabies cases had spread across all nine regencies in Bali (Putra et al. 2013). It was recognized that an understanding of the relationship between dogs and people, along with cultural understanding, was required to make the control program effective. The program needed to include local community knowledge, particularly of behaviours towards dogs. It is clear that a complex relationship between dogs and humans, which contributes to the spread of rabies (Widyastuti et al. 2015). For example, in some regions and ethnic groups, people regard free-roaming or stray dogs as a blessing and keep them in the house as a religious obligation. Many people also believe that washing the wound after a dog bite is unimportant, and instead believe that allowing the dog to lick the wound will help in healing. In addition, (Widyastuti et al. 2015) reported that while most people were supportive of the control program, a subset of the population with specific religious beliefs were opposed to dog culling. An island-wide mass dog vaccination campaign commenced in October 2010 and continued over subsequent years, resulting in a decrease in the number of rabies cases in both dogs and humans (Putra et al. 2013). This could explain why few rabies cases are detected in Indonesia.

In the literature, the Nipah virus is identified as a recently emerged pathogen from the family Paramyxoviridae. The family has two zoonotic viruses, the Hendra virus and the Nipah virus, both classed as henipahviruses. The clinical signs of the Nipah virus in humans include fever, encephalitis, and respiratory and pulmonary disorders, which can lead to death (Ochani et al. 2021). The natural reservoir of the Nipah virus is pteropid bats. A recent report has also demonstrated that the fruit bat species (*Pteropus*) is regarded as the nature reservoir of the Nipah virus, which is a virus of concern for future epidemics and also seems to be spilling over from its animal reservoir to humans (Joshi et al. 2023). The bats do not show clinical signs but may excrete the virus in urine, saliva and blood. Spill-over effects can occur to other species such as pigs and horses, which may then act as intermediate hosts. Transmission may occur through ingestion of contaminated food, direct contact with infected body fluids (human or animal) or through droplet or aerosol exposure. Nipah virus was first identified in Malaysia in 1998 (Chua 2003) when it was associated with an outbreak among pig farmers and abattoir workers. In 2014, a major outbreak of a probable henipavirus was associated with two villages in the Philippines (Ching et al. 2015). A strong association was identified with direct exposure to infected horses. This included exposure to contaminated body fluids during slaughter and/or ­consumption of undercooked horse meat. Human-to-human transmission was also reported. This case study identifies the potential for transmission of pathogens from infected horses (or other spill-over hosts) and people (Ching et al. 2015).

Pathogens associated with zoonotic diseases may be broadly described as bacterial, viral, parasitic or fungal. While viral zoonoses are more commonly considered in large-scale events (ebola, coronaviruses), bacterial and parasitic zoonotic pathogens are implicated commonly on a local scale. Overall, diseases associated with wildlife in this review are giardiasis, leptospirosis, Q fever, ebola virus, rabies virus, West Nile virus and hantavirus. The data from the studies suggested that zoonotic diseases are mostly associated with wildlife in Southeast Asia.

In addition, wildlife has been an important source of infectious diseases transmissible to humans. Some environmental changes drive virus spillover from wildlife, including: (i) Species in global decline because of exploitation and habitat loss share more viruses with people; (ii) Exploitation of wildlife through hunting and the live wild animal trade create the perfect epidemiologic setting for spillover; (iii) Declines in habitat for wild mammals, due to deforestation, development and conversion to cropland, increase disease distribution and animal-human interactions.

It should be noted that a low number of reports on pig diseases were recorded in Southeast Asia. It may come from a few local veterinary journals reporting results that may not be indexed in the databases searched. Besides, relatively few studies were found to evaluate the distribution of both domestic and wildlife diseases in Southeast Asia. Notably, the concept of One Health has become the international standard for zoonotic disease control, which emphasizes a multi-sectoral and transdisciplinary understanding and approach to prevent and mitigate the threat of communicable diseases (Ng et al. 2020). Therefore, further epidemiological investigation using One health approach is necessary to reduce the gaps in disease surveillance and reporting systems as well as to support the prevention and reduction of further outbreaks.

## 5. Conclusion

Taken together, this review provides the current status of emerging zoonotic diseases in Southeast Asia, as well as suggesting the necessary actions for disease control and prevention in the region. Prevention of zoonotic diseases requires general and specific knowledge of pathogens, disease characteristics and transmission. In addition, there is a need for familiarity with potential control measures and the capacity to coordinate activities across human and animal health environments.

## Data Availability

The data that support the findings of this study are available from the corresponding author upon reasonable request.
